# Holmium laser enucleation of the prostate as salvage therapy for benign prostatic obstruction after multiple prior surgical interventions

**DOI:** 10.1007/s00345-026-06539-2

**Published:** 2026-07-03

**Authors:** Hasim Bakbak, Gurpremjit Singh, Ahmad Abdelaziz, Ansh Bhatia, Juanita Velasquez Ospina, Nikita Devi Sharma, Anthony Jose Fuentes, Jonathan E. Katz, Hemendra N. Shah

**Affiliations:** 1 Desai Sethi Urology Institute, University of Miami Miller School of Medicine, Miami, FL USA; 2 Department of Interventional Radiology, University of Miami Miller School of Medicine, Miami, FL USA; 3 University of Miami Miller School of Medicine, Miami, FL USA

**Keywords:** HoLEP, Salvage, Retreatment, Benign prostatic obstruction, Prior BPH surgery, History of BPH surgery

## Abstract

**Introduction:**

Recurrent benign prostatic obstruction (BPO) is a recognized complication following surgery. While Holmium laser enucleation of prostate (HoLEP) has been shown to be safe and effective as a salvage option after single procedure, outcomes in patients with multiple prior interventions remain poorly defined. This study aimed to evaluate the outcomes of HoLEP in patients with multiple prior BPO surgeries compared with surgery-naïve individuals.

**Methods:**

We retrospectively reviewed all patients who underwent HoLEP between July 2017 and August 2025. Patients with two or more previous BPO interventions were identified and matched 1:2 with surgery-naïve controls based on age, prostate size, and anticoagulant/antiplatelet status. Baseline characteristics, postoperative outcomes were compared.

**Results:**

Among 857 patients, 40 with ≥ 2 prior BPO procedures were matched to 72 surgery-naïve controls. Baseline characteristics were largely similar. The control group was significantly frailer with higher modified frailty index scores, lower hemoglobin, higher catheterization rates, and higher residual urine. Both groups experienced significant improvements in voiding. Study group showed more pronounced relief in long term IPSS, but the attrition was high in both groups. Operative time, length of stay, perioperative outcomes, complication rates, and recovery profiles did not differ significantly.

**Conclusions:**

HoLEP appears to be a safe and effective treatment option for patients with a history of multiple BPO procedures, yielding functional outcomes and complication rates comparable to surgery-naive individuals. These findings support HoLEP as a viable salvage strategy even after multiple previous prostate surgeries. Nevertheless, the results should be interpreted considering the study’s limited sample size, attrition, and the possibility of bias.

**Supplementary Information:**

The online version contains supplementary material available at 10.1007/s00345-026-06539-2.

## Introduction

Benign prostatic obstruction (BPO) is a highly prevalent condition in older men, with prevalence rising markedly with advancing age [1]. As longevity increases and the population ages, more patients experience persistent or recurrent lower urinary tract symptoms (LUTS) following surgical management of BPO [2, 3]. Consequently, the clinical demand for repeat BPO interventions is expected to rise.

Transurethral resection of the prostate (TURP) has historically been regarded as the reference standard surgical treatment for benign prostatic obstruction (BPO) and remains widely performed [4, 5]. However, retreatment after TURP is not uncommon, with reoperation rates reported as high as 14.5% in five years [6, 7]. Notably, over the past 2 decades, the surgical landscape for BPO has expanded to include a broad range of minimally invasive surgical therapies (MIST) that can be delivered in outpatient settings and are often attractive to patients because of their convenience and rapid recovery. However, longer-term data suggest that durability may be variable, and a subset of patients experience incomplete or non-durable symptom relief, necessitating escalation of care with surgical retreatment [8, 9].

In contrast, holmium laser enucleation of the prostate (HoLEP) is a size-independent alternative that provides durable symptom relief with low reintervention rates (1–3%) and is endorsed by contemporary AUA and EAU guidelines [4, 5, 10–12]. Despite these advantages, the risk of transient urinary incontinence (TUI) deters patients from selecting HoLEP [13]. Consequently, many patients follow a “step-up” pathway, in which a less invasive option is attempted first, and more definitive surgery is pursued if symptoms persist or recur, thereby increasing the proportion of patients presenting with a prior BPO procedural history when referred for more durable interventions such as HoLEP. In this context, HoLEP has demonstrated safety and efficacy as a salvage procedure following prior interventions, including TURP, photoselective vaporization of the prostate (PVP), prostate artery embolization (PAE), and prostatic urethral lift (UroLift™) [14–16]. 

Although salvage HoLEP series have incidentally included men with multiple previous BPO interventions, no prior study, to our knowledge, has specifically focused on this subgroup with outcomes evaluated against a matched surgery-naïve comparator [14, 15, 17]. To address this gap in the literature, we performed a matched cohort analysis comparing perioperative outcomes and postoperative functional recovery after HoLEP in patients with a history of two or more prior BPO procedures versus surgery-naïve controls.

## Methods

### Study design

Patients who underwent HoLEP by a single surgeon (HNS) at a tertiary academic center between July 2017 and August 2025 were retrospectively identified using a prospectively maintained institutional HoLEP registry. Patients with multiple prior BPO interventions were identified as the study cohort and matched 1:2 with surgery-naïve controls based on age, prostate size, and anticoagulant/antiplatelet status and was conducted using the nearest-neighbor method. Patients were included in the study cohort if HoLEP was performed for recurrent or persistent BPO after two or more prior BPO interventions, including cases related to prostatic regrowth, incomplete treatment, or persistent obstruction. Time intervals between HoLEP and prior procedures were calculated. Exclusion criteria included neurogenic bladder, urethral stricture, bladder neck contracture or sclerosis, bladder malignancy, and a history of locally advanced prostate cancer; patients undergoing HoLEP as part of multimodal management prior to radiotherapy or high-intensity focused ultrasound for organ-confined prostate cancer were also excluded. The study is approved by the institutional review board (IRB #20180511) and reported in accordance with the Strengthening the Reporting of Observational Studies in Epidemiology (STROBE) guidelines for cohort studies [18]. 

### Perioperative management and surgical technique

Prostate size was determined on imaging obtained within 12 months before surgery. Elevated PSA was assessed with 4Kscore, multiparametric prostate magnetic resonance imaging (mpMRI), and/or biopsy based on shared decision-making. Anticoagulant and antiplatelet agents were typically held preoperatively in coordination with the patient’s primary care physician or cardiologist unless temporary discontinuation was contraindicated. A urine culture was obtained within two weeks of the procedure and patients with positive cultures received culture-directed antibiotics prior to surgery.

All HoLEP cases were performed under general anesthesia as preferred by our anesthesiologist in the lithotomy position using en-bloc enucleation technique as previously described [19]. Recently, we modified our surgical template with an attempt to preserve anterior fibromuscular stroma and overlying mucosa when possible. One high-volume attending surgeon performed or directly supervised all procedures, with resident and fellow participation. Enucleation was completed using a 26-French laser resectoscope (Karl Storz, Tuttlingen, Germany) with a 550-µm laser fiber connected to a 100-W or 120-W holmium: YAG platform (with Moses pulse modulation when available), using typical settings of 2 J and 30 Hz. Enucleated adenoma was morcellated intravesically using a mechanical morcellator (e.g., VersaCut™ or Piranha). The technique was further modified based on the morphology of prostate regrowth in the study group [20]. In 4 patients with prior UroLift System implants, urethral end plates and capsular tabs encountered during enucleation were ablated using laser energy. When identified during morcellation, these components were removed separately to minimize the risk of morcellator blade damage. At the end of the case, a three-way Foley catheter was placed (typically 22-French) without traction, and continuous bladder irrigation was initiated. Patients were admitted overnight for observation. On postoperative day 1 (POD 1), complete blood count and basic metabolic profile was obtained, followed by a voiding trial. Patients who voided adequately were discharged. Those who failed the trial or had PVR > 150 mL were discharged with a catheter and scheduled for an outpatient repeat trial within 2–7 days. Patients with chronic painless retention with PVR > 500 mL who were not catheter-dependent preoperatively, were typically discharged with an indwelling Foley catheter for 1–3 weeks. All prostate-directed medications were discontinued immediately after surgery.

### Postoperative follow-up and outcomes

Postoperative assessments were performed at 6 weeks and at 3, 6, and 12 months. Functional outcomes included International Prostate Symptom Score (IPSS), maximum urinary flow rate (Qmax), and post-void residual urine volume (PVR). PSA nadir was recorded at approximately 3 months when available. Postoperative complications including blood transfusion requirement, urinary retention, urinary tract infection, urethral stricture, bladder-neck contracture, and urinary incontinence were captured. Urinary incontinence was defined as any involuntary leakage requiring pad use at any postoperative time point.

### Data collection and study variables

Baseline variables included age, race/ethnicity, BMI, comorbidities, hemoglobin, creatinine, catheterization, prostate size, PVR, urine culture, indication, and history of PCa. Frailty was quantified using the modified frailty index (mFI-11), derived from the Canadian Study of Health and Aging Frailty Index, and validated across various surgical populations [21]. For the study cohort, prior BPO procedural history was characterized by number, type, and temporal sequence of prior interventions. Perioperative variables included operative time, enucleated tissue weight, enucleation efficiency, length of stay, catheter duration, and POD 1 laboratory values. Postoperative outcomes included complications graded according to the Clavien-Dindo classification and functional follow-up parameters, including IPSS, Qmax, PVR, PSA, and follow-up duration.

### Statistical analysis

Continuous variables were summarized as means with standard deviations or as medians with interquartile ranges, based on data distribution, and were compared using independent-samples t-tests or Wilcoxon signed rank tests as appropriate. Categorical variables were compared using chi-square or Fisher exact tests as appropriate.

To minimize selection bias, a matched case-control analysis was performed. Patients undergoing salvage procedures were matched to control patients in a targeted 1:2 ratio using nearest neighbor matching. Matching criteria included exact matching for anticoagulant/antiplatelet use and approximate matching for continuous variables. We used a maximum caliper width of 0.2 SD of logit score. A suitable second control for 8 cases was not found therefore for these 8 cases, a 1:1 match was retained to prevent introduction of poorly matched controls, resulting in a final matched cohort of 40 cases and 72 controls (Effective match ratio of 1:1.8).

Given the higher baseline frailty, greater catheter dependency, lower hemoglobin, and higher preoperative PVR observed in the control cohort, a multivariable sensitivity analysis was performed to isolate the independent effect of prior BPO surgery on outcomes. Multiple linear and binary logistic regression models were utilized, adjusting for these specific baseline disparities.

A two-sided p-value < 0.05 was considered statistically significant. Statistical analyses were performed using R version 4.3.1 (R Foundation for Statistical Computing, Vienna, Austria). Missing data were addressed through available-case analysis, with denominators reported for each outcome. We generated a swimmer plot to visualize prior BPO surgeries.

## Results

Among 857 HoLEP patients, 40 (4.7%) met inclusion criteria and were compared with 72 (8.4%) matched surgery-naïve controls. Baseline characteristics were largely similar. The control group was significantly frailer than the study cohort, both in terms of the proportion of frail patients and mean frailty burden (29.2% vs. 10.0%, *p* = 0.031; mFI 0.26 vs. 0.17, *p* = 0.027). Surgery-naive patients also had significantly higher rates of catheter dependency and lower preoperative hemoglobin levels (36.1% vs. 17.5%, *p* = 0.038; 13.56 vs. 14.18 g/dL, *p* = 0.046). In contrast, the study cohort had significantly lower preoperative PVR volumes (143.92 vs. 248.78 mL, *p* = 0.033). Among patients in the study group, a total of 89 prior procedures were identified, including TURP, PVP, PAE, UroLift, and water vapor thermal therapy (Rezum). The most recent and second-most-recent procedures were predominantly TURP, Greenlight, and PAE (Table 1). The swimmer plot demonstrates that TURP and PVP were often followed by a longer interval before subsequent BPO therapy, whereas newer approaches tend to cluster closer to HoLEP (Supplementary Fig. 1).

**Table 1:** Comparison of baseline and demographic characteristics between surgery-naïve patients and patients with multiple prior BPO procedures

	Study group (*N* = 40)	Control group (*N* = 72)	*P* value
*Baseline characteristics*			
Age (Years)	72.08 ± 7.43	72.32 ± 7.74	0.871
*Race*			0.204
White/Caucasian	37 (92.5%)	57 (79.2%)	
Black/African American	1 (2.5%)	8 (11.1%)	
Asian	2 (5.0%)	4 (5.6%)	
*Ethnicity*			0.290
Non-Hispanic/Latino	24 (60%)	34 (47.2%)	
Hispanic/Latino	16 (40%)	36 (50%)	
BMI (kg/m²)	26.42 ± 4.62	27.01 ± 4.33	0.504
HTN	22 (55.0%)	49 (68.1%)	0.169
DM	7 (17.5%)	20 (27.8%)	0.223
Frail (mFI ≥ 0.36)	4 (10%)	21 (29.2%)	**0.013**
Frailty index	0.17 ± 0.18	0.26 ± 0.21	**0.027**
Hemoglobin (g/dL)	14.18 ± 1.33	13.56 ± 1.63	**0.046**
Creatinine (mg/dL)	1.06 ± 0.30	1.10 ± 0.29	0.545
Anticoagulant/antiplatelet	19 (47.5%)	34 (47.2%)	0.977
Prostate size (cc)	108.50 ± 71.55	108.31 ± 68.43	0.99
PVR	143.82 ± 213.64	248.78 ± 225.33	**0.033**
PVR > 300	6 (15%)	17 (23.6%)	0.213
Catheter dependency	7 (17.5%)	26 (36.1%)	**0.038**
Performing CIC	4 (10.0%)	6 (8.3%)	0.767
Positive urine culture	4 (10%)	15 (20.8%)	0.192
*Prior BPO surgeries*			
Number of prior surgeries	Total: 89 surgeries 2 Patients x4 5 Patients x3 33 Patients x2	0	NA
Prior TURP	42	0	NA
Prior greenlight photovaporization	20	0	NA
Prior PAE	16	0	NA
Prior UroLift	4	0	NA
Prior TUMT	3	0	NA
Prior Rezum	1	0	NA
Prior Thulep	1	0	NA
Prior TUIP	1	0	NA
Prior TUNA	1	0	NA
Gap between HoLEP-most recent BPO surgery (years)	3.74 (3.98)	NA	NA
Gap between HoLEP-2nd most recent BPO surgery (years)	10.6 (8.14)	NA	NA
Gap between most recent-2nd most recent BPO surgery (years)	6.88 (7.6)	NA	NA
Latest BPO surgery before HoLEP	12x PAE 13x TURP 10x GreenLight 1x Rezum 1x UroLift 1x Thulep 1x TUIP 1x TUMT	NA	NA
Second most recent BPO surgery	22x TURP 10x GreenLight 4x PAE 3x UroLift 1x TUMT	NA	NA
Third most recent BPO surgery	5x TURP 1x TUNA 1x TUMT	NA	NA
Fourth most recent BPO surgery	2x TURP	NA	NA
*PCa Workup*			
PSA (ng/mL)	4.87 ± 5.00	6.44 ± 6.04	0.167
History of GG1 Pca (Active Surveillance)	5 (12.5%)	9 (12.5%)	
*Indications #*			
Recurrent urinary retention	20 (50.0%)	43 (59.7%)	0.32
Recurrent gross hematuria	7 (17.5%)	8 (11.1%)	0.341
Recurrent UTI	9 (22.5%)	10 (13.9%)	0.245
Bothersome LUTS	28 (70.0%)	56 (77.8%)	0.362

#- some patients had multiple indications for surgery

[bold] typing character refers to achieved statistical significance defined as p<0.05

Perioperative characteristics were comparable between groups. Operative time, resected volume, and length of stay were similar, with no intraoperative complications. The POD 1 creatinine and hemoglobin drop were similar. Catheter duration did not differ significantly between the groups. iPCa rates were similar, including a small number with known disease preoperatively. Both groups demonstrated substantial functional improvement in IPSS, Qmax and PVR. Baseline IPSS was comparable between groups. At 12 months, the study group demonstrated lower IPSS scores compared with controls (1.78 vs. 8.00, *p* = 0.007); however, this analysis was limited by small sample sizes with available symptom-score data (study group: *n* = 9; control group: *n* = 4). IPSS outcomes at 3 and 6 months were otherwise comparable between groups (Table 2).

**Table 2:** Comparison of HoLEP outcomes between surgery-naïve patients and patients with multiple prior BPO procedures

	Study group (*N* = 40)		Control group (*N* = 72)		*P* value
*Intraoperative*					
Operative time (minutes)	142.15 ± 79.88		150.00 ± 69.83		0.589
Enucleated tissue weight (grams)	63.45 (32.85–95.75)		63.0 (44.0–96.4)		0.605
Enucleation efficiency (g/min)	0.52 ± 0.34		0.54 ± 0.33		0.755
*Postoperative*					
Creatinine POD 1 (mg/dL)	1.07 ± 0.28		1.04 ± 0.29		0.624
Hgb POD1 (g/dL)	11.35 ± 3.64		10.83 ± 3.90		0.488
Hgb drop POD 1 (g/dL)	2.53 ± 2.86		2.73 ± 3.76		0.752
Length of stay (days)	1.15 ± 0.62		1.38 ± 0.96		0.135
Duration of catheter (days)	4.98 ± 7.30		5.49 ± 7.97		0.732
PSA at 3 months (ng/mL)	0.91 ± 1.99		0.49 ± 0.40		0.131
Incidental prostate cancer	8 (20.0%)		12 (16.7%)		0.659
*IPSS*	*N*	*Study group*	*N*	*Control group*	
Baseline	35	21.03 ± 6.55	59	19.14 ± 7.90	0.235
3 months	29	4.90 ± 3.98	45	6.53 ± 6.20	0.211
6 months	14	3.43 ± 7.52	3	5.00 ± 1.00	0.73
12 months	9	1.78 ± 1.64	4	8.00 ± 5.42	**0.007**
*Qmax (mL/s)*	*N*	*Study group*	*N*	*Control group*	
Baseline	31	9.15 ± 4.98	48	7.78 ± 4.63	0.216
3 months	31	14.99 ± 7.34	49	17.50 ± 11.76	0.29
6 months	16	18.08 ± 11.59	10	13.04 ± 9.14	0.256
12 months	16	15.19 ± 9.96	5	11.54 ± 4.85	0.445
*PVR (mL)*	*N*	*Study group*	*N*	*Control group*	
Baseline	34	143.82 ± 213.64	54	248.78 ± 225.33	**0.033**
3 months	32	36.28 ± 55.24	53	45.55 ± 86.81	0.59
6 months	16	32.06 ± 66.61	11	40.64 ± 52.94	0.725
12 months	17	27.18 ± 26.41	5	52.40 ± 36.94	0.101
*Complications*					
Major (CD ≥ 3)	3 (7.5%)		2 (2.8%)		0.694
Bladder neck contracture	1 (2.5%)		1 (1.4%)		0.671
Urethral stricture	1 (2.5%)		1 (1.4%)		0.671
Minor (CD ≤ 2)	18 (45.0%)		32 (44.4%)		0.694
Transfusion	1 (2.5%)		2 (2.8%)		0.93
Failed voiding trial on POD1	5 (12.5%)		3 (4.2%)		0.101
UTI requiring antibiotics	3 (7.5%)		1 (1.4%)		0.095
Transient incontinence (6 weeks)	16 (41.0%)		28 (39.4%)		0.871
Incontinence at 1 year	1 (2.5%)		2 (2.8%)		1
Duration of follow up	16.87 ± 19.24		7.49 ± 9.44		-

[bold] typing character refers to achieved statistical significance defined as p<0.05

Overall major (CD ≥ 3) and minor (CD ≤ 2) complication rates were low and similar between groups. There were 3 (7.5%) major and 18 (45%) minor complications in the study group, compared with 2 (2.8%) major and 32 (44.4%) minor complications in the control group. Among the two patients in the control group who received a transfusion, one had preoperative anemia. Bladder neck contracture occurred at comparable rates between groups.

Due to baseline cohort differences in frailty, preoperative catheter dependency, hemoglobin, and PVR, a multivariable sensitivity analysis was performed to isolate the independent effect of prior BPO surgery on outcomes. Multiple linear and binary logistic regression models were utilized, adjusting for these specific baseline disparities. Following risk adjustment, a history of multiple prior BPO procedures was not an independent predictor of major complications (*p* = 0.854; aOR 0.78). Furthermore, multivariable adjustment confirmed no statistically significant difference in total operative time (*p* = 0.644) or IPSS at 12 months (Supplementary Tables 1, 2).

## Discussion

The need for surgical reintervention is a well-recognized long-term complication across all surgical treatments for BPO. Progressive prostate growth with aging is commonly implicated as a key factor contributing to recurrent BPO [22]. In addition, with the expanding adoption of MISTs, a subset of patients experiences incomplete or non-durable symptom relief and subsequently pursue more definitive surgical management [8, 9]. Collectively, these trends are expected to increase the number of patients presenting with multiple prior BPO interventions. To our knowledge, our study is the first to examine this focused subgroup of patients with multiple prior BPO interventions. HoLEP provided safe, effective relief after multiple prior BPO procedures, extending prior salvage series [14–17]. 

Baseline demographics and preoperative characteristics were mostly comparable between groups. Surgery-naive patients were more vulnerable at baseline, with greater frailty, lower hemoglobin, and higher rates of catheter dependency. This likely reflects an inherent referral-pattern bias. Considering our institution’s collaboration with community hospitals and the suitability of HoLEP for catheter-dependent patients, the surgery-naive cohort was likely affected by referral-pattern selection bias. The lower hemoglobin in the surgery-naive cohort likewise reflects a greater chronic disease burden. These findings suggest that prior surgical history should not be interpreted as a sole surrogate for overall fitness and underscore the potential for selection bias despite matched analyses. The study group had comparable baseline IPSS aligning with Durant et al [8]. Similar to Marien et al. and Durant et al., we observed lower baseline PVR in the study cohort [8, 14]. We hypothesize that the relatively good bladder emptying observed in the study group might be due to delay in decompensatory phase of bladder outlet obstruction related to relief of obstruction achieved by earlier first two surgical interventions in study group. This is consistent with improved urodynamic parameters after de-obstruction [23]. 

Another notable finding was the temporal pattern between prior interventions. We found that the interval between the most recent procedure and HoLEP was shorter than the interval separating earlier treatments. This pattern may reflect differences in durability between procedural eras: traditional operations such as TURP typically achieve greater tissue removal and more sustained symptom relief, whereas newer minimally invasive techniques, despite their appeal due to lower perioperative morbidity and procedural convenience, may offer less durable outcomes [17, 24]. This issue is particularly salient in the retreatment setting, where failure of an initial BPO intervention often necessitates escalation to a more definitive surgical strategy. Beyond clinical considerations, the cumulative psychological burden of repeated procedures, as well as their associated financial impact, should be incorporated into treatment decision making [25]. Consistent with this interpretation, the swimmer plot visually illustrates a durability gradient, with TURP and PVP generally associated with longer intervals before subsequent BPO therapy, while newer modalities tend to cluster closer to HoLEP (Supplementary Fig. 1). Collectively, these findings support the use of definitive enucleation approaches when symptoms recur after prior BPO treatment [26]. 

Although technique modifications may be needed in altered anatomy including removal of urethral end plates and capsular tabs of prior UroLift System implants, this did not increase operating room time [20, 27]. The safety and reproducibility of laser enucleation have been supported by contemporary studies evaluating refinements in HoLEP technique and comparisons across enucleation platforms. For example, HoLEP performed with Virtual Basket mode has been associated with improved hemostatic control and procedural efficiency, while prospective multicenter randomized data comparing HoLEP and ThuLEP have demonstrated favorable perioperative and functional outcomes for laser enucleation approaches. These findings support the broader role of endoscopic enucleation as a reproducible and effective strategy for BPO management, including in technically complex settings such as salvage surgery after prior interventions [28, 29]. Consistent with prior salvage HoLEP series following a single prior BPO intervention, we observed no meaningful differences in length of stay compared with surgery-naïve controls, supporting preserved perioperative efficiency even in re-operative settings [17, 30]. Both groups demonstrated substantial postoperative functional improvement compared with baseline mirroring Marien et al. and Khater et al., who reported preserved efficacy of salvage HoLEP after prior TURP, PVP, PAE, or UroLift [14, 20]. The study group demonstrated greater improvement in long-term IPSS; however, this finding should be interpreted cautiously due to attrition bias and limited symptom-score availability at 12 months, particularly among controls. Given that IPSS outcomes at 3 and 6 months were comparable between groups, these earlier time points may provide a more reliable assessment of functional recovery. Accordingly, the observed 12-month IPSS difference should be considered exploratory rather than definitive.

In line with prior salvage HoLEP series, overall complication rates were low. Oh et al. reported no major surgical complications in salvage cases and found comparable perioperative outcomes versus primary HoLEP, while Marien et al. similarly observed no difference in transfusion risk in retreatment patients [14, 30]. In a post-TURP population, Khater et al. similarly demonstrated that HoLEP did not confer a higher risk of major complications [20]. Although not statistically significant, failed POD1 voiding trial and UTI requiring antibiotics were more frequent in the study group. These trends may reflect altered anatomy, prior instrumentation, or greater perioperative complexity in patients with multiple interventions. However, given the small sample size and lack of statistical significance, these findings should be interpreted cautiously and evaluated in larger studies. Taken together, our data extend these reassuring safety signals to an even more challenging population suggesting that prior instrumentation and prior outlet surgery do not necessarily translate into a “higher-risk”.

Patterns in specific postoperative events provide additional context. TUI rates were similar consistent with the literature reporting similar TUI rates between primary HoLEP and salvage HoLEP [17]. Additionally, urethral stricture and bladder neck contracture were rare and comparable between groups suggesting that HoLEP can be performed without an excess of these late complications in a multi-procedure setting. Prior retreatment series similarly report low absolute postoperative stricture rates, albeit with a modest increase in some cohorts [14]. In our cohort, iPCa rates were comparable and are consistent with prior HoLEP literature reporting incidence, ranging from 5.64 to 23.3% [31, 32].

Lastly, our study addresses a common clinical hesitation among urologists regarding the role of HoLEP in patients who have undergone multiple prior interventions. There is often concern that repeated transurethral surgeries may lead to distorted anatomy, urethral compromise, and technically challenging endoscopic conditions that could predispose to inferior outcomes or even procedural failure. Despite substantial prior instrumentation, functional recovery, perioperative safety, and complication rates were comparable to those in surgery-naïve patients, supporting HoLEP as a definitive option even after multiple earlier procedures.

### Limitations

This study is limited by its retrospective, single-center design, and the small sample size of the multi-procedure cohort, which may restrict generalizability and limit power to detect small differences between groups. We did not use ASA class and Charlson Comorbidity Index in our study as the information about same was not consistently available in our database. Another limitation is that morcellation efficiency could not be evaluated, as morcellation time was not consistently recorded in our retrospective registry despite its potential relevance given the possible altered tissue characteristics that may occur following multiple prior BPO procedures. It is likely that number of prior surgeries might influence the outcome of salvage HoLEP. However, only seven patients in our study had three or more prior BPO procedures. Hence the study was underpowered to determine whether outcomes differed according to the exact number of prior interventions. The heterogeneity of prior interventions introduces intrinsic variability that may confound perioperative and functional outcomes. Another limitation of this study is that, although the study group included patients with both ablative and non-ablative prior procedures, the small sample size within each subgroup limited the ability to determine whether outcomes differed by prior procedure type. Follow-up duration was shorter in the surgery-naïve control cohort, likely reflecting the retrospective design and variability in clinical follow-up. This imbalance limits comparison of long-term outcomes, particularly 12-month IPSS, and may introduce attrition bias. Therefore, longer-term functional comparisons should be interpreted cautiously. In addition, longitudinal follow-up was incomplete. Despite these limitations, our findings provide evidence that HoLEP can effectively salvage recurrent BPO in patients with multiple prior BPO procedures.

## Conclusion

HoLEP appears to be a safe and effective treatment option for patients with a history of multiple prior BPO procedures, yielding functional outcomes and complication rates comparable to surgery-naive individuals. These findings support the role of HoLEP as a viable salvage strategy even after multiple previous prostate surgeries. Nevertheless, the results should be interpreted considering the study’s limited sample size and the possibility of inherent selection bias despite matched analysis. As an early study to evaluate this specific context, our findings provide a foundation for future studies.

## Supplementary Information

Below is the link to the electronic supplementary material.

Supplementary Material 1

Supplementary Material 2

## Data Availability

The data that support the findings of this study are available but restrictions apply to the availability of these data, and are not publicly available. The data are, however, available upon request and with the permission.

## References

[1] Berry SJ, Coffey DS, Walsh PC, Ewing LL (1984) The development of human benign prostatic hyperplasia with age. J Urol 132:474–479. 10.1016/S0022-5347(17)49698-4 6206240 10.1016/s0022-5347(17)49698-4

[2] Kontis V, Bennett JE, Mathers CD, Li G, Foreman K, Ezzati M (2017) Future life expectancy in 35 industrialised countries: projections with a Bayesian model ensemble. Lancet 389:1323–1335. 10.1016/S0140-6736(16)32381-9 28236464 10.1016/S0140-6736(16)32381-9 PMC5387671

[3] Kim A, Hak AJ, Choi WS, Paick SH, Kim HG, Park HK (2021) Comparison of long-term effect and complications between holmium laser enucleation and transurethral resection of prostate: nations-wide health insurance study. Urology 154:300–307. 10.1016/j.urology.2021.04.019 33933503 10.1016/j.urology.2021.04.019

[4] Gravas S, Gacci M, Gratzke C et al (2023) Summary paper on the 2023 European Association of Urology guidelines on the management of non-neurogenic male lower urinary tract symptoms. Eur Urol 84:207–222. 10.1016/j.eururo.2023.04.008 37202311 10.1016/j.eururo.2023.04.008

[5] Lerner LB, McVary KT, Barry MJ et al (2021) Management of lower urinary tract symptoms attributed to benign prostatic hyperplasia: AUA guideline part II-surgical evaluation and treatment. J Urol 206:818–826. 10.1097/JU.0000000000002184 34384236 10.1097/JU.0000000000002184

[6] Rassweiler J, Teber D, Kuntz R, Hofmann R (2006) Complications of transurethral resection of the prostate (TURP)-incidence, management, and prevention. Eur Urol 50:969–979. 10.1016/j.eururo.2005.12.042 16469429 10.1016/j.eururo.2005.12.042

[7] Lourenco T, Pickard R, Vale L et al (2008) Alternative approaches to endoscopic ablation for benign enlargement of the prostate: systematic review of randomised controlled trials. BMJ 337:a449. 10.1136/bmj.39575.517674.BE 18595932 10.1136/bmj.39575.517674.BE PMC2443595

[8] Durant AM, Moore J, Voleti S et al (2022) Salvage versus primary holmium laser enucleation of the prostate: trends, outcomes and safety analysis. World J Urol 40:2305–2312. 10.1007/s00345-022-04098-w 35867143 10.1007/s00345-022-04098-w

[9] Parmar M, Katz JE, Blachman-Braun R et al (2021) Safety and efficacy of holmium laser enucleation of prostate as salvage procedure for persistent or recurrent lower urinary tract symptoms secondary to bladder outlet obstruction after prior prostate artery embolization: a match analysis. World J Urol 39:4199–4206. 10.1007/s00345-021-03747-w 34081181 10.1007/s00345-021-03747-w

[10] Tan AH, Gilling PJ, Kennett KM, Frampton C, Westenberg AM, Fraundorfer MR (2003) A randomized trial comparing holmium laser enucleation of the prostate with transurethral resection of the prostate for the treatment of bladder outlet obstruction secondary to benign prostatic hyperplasia in large glands (40 to 200 grams). J Urol 170:1270–1274. 10.1097/01.JU.0000086948.55973.00 14501739 10.1097/01.ju.0000086948.55973.00

[11] Krambeck AE, Handa SE, Lingeman JE (2010) Experience with more than 1,000 holmium laser prostate enucleations for benign prostatic hyperplasia. J Urol 183:1105–1109. 10.1016/j.juro.2009.11.034 20092844 10.1016/j.juro.2009.11.034

[12] Elmansy HM, Kotb A, Elhilali MM (2011) Holmium laser enucleation of the prostate: long-term durability of clinical outcomes and complication rates during 10 years of followup. J Urol 186:1972–1976. 10.1016/j.juro.2011.06.065 21944127 10.1016/j.juro.2011.06.065

[13] Houssin V, Olivier J, Brenier M et al (2021) Predictive factors of urinary incontinence after holmium laser enucleation of the prostate: a multicentric evaluation. World J Urol 39:143–148. 10.1007/s00345-020-03169-0 32219512 10.1007/s00345-020-03169-0

[14] Marien T, Kadihasanoglu M, Tangpaitoon T et al (2017) Outcomes of holmium laser enucleation of the prostate in the re-treatment setting. J Urol 197:1517–1522. 10.1016/j.juro.2016.12.098 28043843 10.1016/j.juro.2016.12.098

[15] Zakaria AS, Hodhod A, Abbas L et al (2022) Outcomes of top-down holmium laser enucleation of prostate for recurrent/residual benign prostatic hyperplasia: one-year follow-up. Adv Urol 2022:5185114. 10.1155/2022/5185114 36247205 10.1155/2022/5185114 PMC9553753

[16] Mokhtarzadehazar P, Ferguson B, Davis G, El Tayeb M (2025) Feasibility of holmium laser enucleation of the prostate (HoLEP) after prior prostate intervention for benign prostatic hyperplasia (BPH). Urology. 10.1016/j.urology.2025.11.248 41360301 10.1016/j.urology.2025.11.248

[17] Jaeger CD, Krambeck AE (2013) Holmium laser enucleation of the prostate for persistent lower urinary tract symptoms after prior benign prostatic hyperplasia surgery. Urology 81:1025–1029. 10.1016/j.urology.2013.01.019 23465142 10.1016/j.urology.2013.01.019

[18] von Elm E, Altman DG, Egger M, Pocock SJ, Gøtzsche PC, Vandenbroucke JP, STROBE Initiative (2007) Strengthening the Reporting of Observational Studies in Epidemiology (STROBE) statement: guidelines for reporting observational studies. Ann Intern Med 147:573–577. 10.7326/0003-4819-147-8-200710160-00010 17938396 10.7326/0003-4819-147-8-200710160-00010

[19] Martos M, Katz JE, Parmar M et al (2021) Impact of perioperative factors on nadir serum prostate-specific antigen levels after holmium laser enucleation of prostate. BJUI Compass 2:202–210. 10.1002/bco2.68 35475131 10.1002/bco2.68 PMC8988639

[20] Khater U, Smith NA, Katz JE et al (2022) Does prior transurethral resection of prostate have a negative impact on the outcome of holmium laser enucleation of prostate? Results from a prospective comparative study. Urol Ann 14:118–124. 10.4103/ua.ua_106_21 35711492 10.4103/ua.ua_106_21 PMC9197008

[21] Velanovich V, Antoine H, Swartz A, Peters D, Rubinfeld I (2013) Accumulating deficits model of frailty and postoperative mortality and morbidity: its application to a national database. J Surg Res 183:104–110. 10.1016/j.jss.2013.01.021 23415494 10.1016/j.jss.2013.01.021

[22] Williams AM, Simon I, Landis PK et al (1999) Prostatic growth rate determined from MRI data: age-related longitudinal changes. J Androl 20:474–480. 10.1002/j.1939-4640.1999.tb02545.x 10452590

[23] Akshay A, Hashemi Gheinani A, Besic M et al (2024) De-obstruction of bladder outlet in humans reverses organ remodelling by normalizing the expression of key transcription factors. BMC Urol 24:33. 10.1186/s12894-024-01417-8 38326801 10.1186/s12894-024-01417-8 PMC10848355

[24] Heiman J, Snead WM, DiBianco JM (2024) Persistent lower urinary tract symptoms after BPH surgery. Curr Urol Rep 25:125–131. 10.1007/s11934-024-01202-y 38578550 10.1007/s11934-024-01202-y

[25] Sanchez DE, Ghoreifi A, Storino Ramacciotti L et al (2025) The safety and feasibility of Aquablation in patients with previous surgery for benign prostatic hyperplasia. J Endourol 39:50–55. 10.1089/end.2024.0370 39639800 10.1089/end.2024.0370

[26] Maheshwari PN, Kolsawala HG (2025) Aquablation or anatomic endoscopic enucleation of prostate. J Endourol 39:56. 10.1089/end.2024.0855 39823573 10.1089/end.2024.0855

[27] McAdams S, Funk JT, Navetta AF et al (2017) Holmium laser enucleation of the prostate after prostatic urethral lift surgery: feasibility and technical considerations from a multi-institutional case series. J Endourol 31:774–779. 10.1089/end.2017.0224 28586247 10.1089/end.2017.0224

[28] Rocco B, Sighinolfi MC, Sandri M et al (2021) Holmium laser enucleation of the prostate with Virtual Basket mode: faster and better control on bleeding. BMC Urol 21:28. 10.1186/s12894-021-00797-5 33622326 10.1186/s12894-021-00797-5 PMC7903737

[29] Bozzini G, Berti L, Aydoğan TB et al (2021) A prospective multicenter randomized comparison between Holmium Laser Enucleation of the Prostate (HoLEP) and Thulium Laser Enucleation of the Prostate (ThuLEP). World J Urol 39:2375–2382. 10.1007/s00345-020-03468-6 32997262 10.1007/s00345-020-03468-6

[30] Oh JK, Bae J, Jeong CW, Paick JS, Oh SJ (2014) Salvage holmium laser enucleation of prostate to treat residual benign prostatic hyperplasia. Can Urol Assoc J 8:E235–E240. 10.5489/cuaj.1494 24839489 10.5489/cuaj.1494 PMC4001650

[31] Porto JG, Blachman-Braun R, Ajami T et al (2024) Incidental prostate cancer after holmium laser enucleation of the prostate: critical analysis of independent risk factors and impact on surgical outcomes. BJUI Compass 5:374–381. 10.1002/bco2.306 38481670 10.1002/bco2.306 PMC10927913

[32] Yilmaz M, Toprak T, Suarez-Ibarrola R, Sigle A, Gratzke C, Miernik A (2022) Incidental prostate cancer after holmium laser enucleation of the prostate: a narrative review. Andrologia 54:e14332. 10.1111/and.14332 34837229 10.1111/and.14332

