# Supplementary figures and images for: Holmium laser enucleation of the prostate as salvage therapy for benign prostatic obstruction after multiple prior surgical interventions
Source: World J Urol. 2026 Jul 3;44(1):479. doi: 10.1007/s00345-026-06539-2 (PMC13331870; doi:10.1007/s00345-026-06539-2)

**Figure 1. Timeline of prior BPO interventions preceding HoLEP**


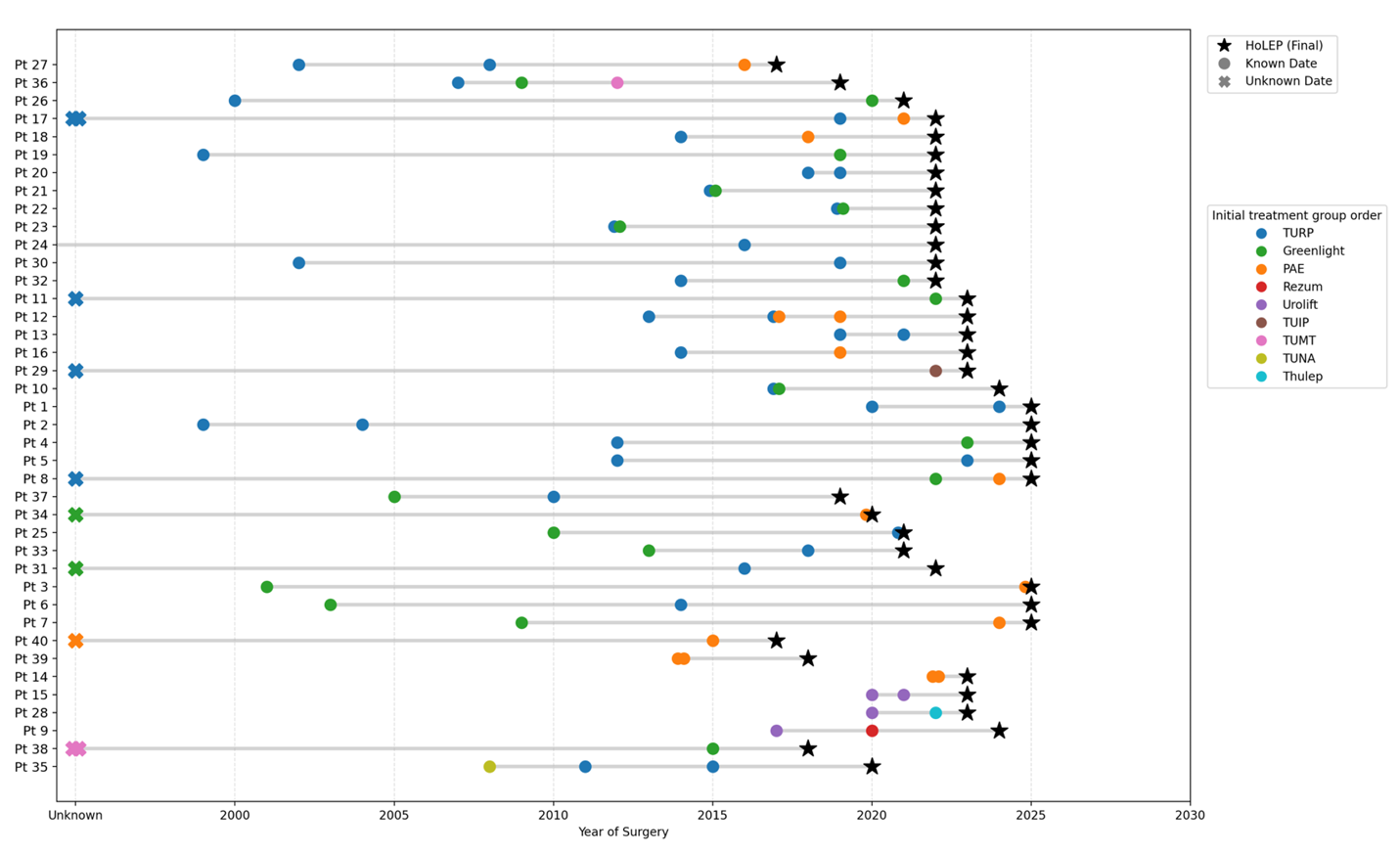

Supplement: Supplementary file 1 — Supplementary Material 1 [file 345_2026_6539_MOESM1_ESM.docx]
