# Supplementary material for: Holmium laser enucleation of the prostate as salvage therapy for benign prostatic obstruction after multiple prior surgical interventions
Source: World J Urol. 2026 Jul 3;44(1):479. doi: 10.1007/s00345-026-06539-2 (PMC13331870; doi:10.1007/s00345-026-06539-2)
Supplement: Supplementary file 2 — Supplementary Material 2 [file 345_2026_6539_MOESM2_ESM.docx]

**Supplementary table1: Multivariate linear regression models for continuous outcome baseline imbalances:**

| **Variable** | **Unstandardized Coefficient (B)** | **Standard Error** | **P-Value** |
| --- | --- | --- | --- |
| **Model 1: IPSS at 1 Year** |  |  |  |
| Study Group (Matched) | -8.377 | 4.332 | 0.094 |
| Frailty (mFI-11 Score) | 1.967 | 2.515 | 0.460 |
| Catheter Dependency | -2.221 | 4.48 | 0.635 |
| Preoperative Hemoglobin | 0.501 | 0.855 | 0.577 |
| Preoperative PVR | -0.01 | 0.006 | 0.159 |
| **Model 2: Operative Time (Minutes)** |  |  |  |
| Study Group (Matched) | -8.297 | 17.909 | 0.644 |
| Frailty (mFI-11 Score) | -3.668 | 8.395 | 0.663 |
| Catheter Dependency | -19.163 | 21.62 | 0.378 |
| Preoperative Hemoglobin | 4.341 | 5.953 | 0.468 |
| Preoperative PVR | -0.004 | 0.037 | 0.922 |

**Supplementary table 2: Multivariate logistic regression model for major complications:**

| **Variable** | **Adjusted Odds Ratio (aOR)** | **Standard Error (S.E.)** | **P-Value** |
| --- | --- | --- | --- |
| Study Group (Matched) | 0.78 | 1.349 | 0.854 |
| Frailty (mFI-11 Score) | 0.739 | 0.691 | 0.661 |
| Catheter Dependency | 2.755 | 1.36 | 0.456 |
| Preoperative Hemoglobin | 0.965 | 0.445 | 0.937 |
| Preoperative PVR | 1 | 0.003 | 0.88 |
